# Enabling in vivo comparisons of different four-dimensional magnetic resonance imaging sequences for radiotherapy guidance using visual biofeedback

**DOI:** 10.1016/j.phro.2025.100815

**Published:** 2025-08-05

**Authors:** Katrinus Keijnemans, Tim Schakel, Bastien Lecoeur, Pim T.S. Borman, William A. Hall, Bas W. Raaymakers, Andreas Wetscherek, Eric S. Paulson, Martin F. Fast

**Affiliations:** a Department of Radiotherapy, University Medical Center Utrecht, Heidelberglaan 100, Utrecht, 3584 CX, The Netherlands; b Joint Department of Physics at The Institute of Cancer Research and The Royal Marsden NHS Foundation Trust, 15 Cotswold Rd, London, SM2 5NG, United Kingdom; c Department of Computing at Imperial College London, 180 Queen’s Gate, London, SW7 2RH, United Kingdom; d Department of Radiation Oncology, Medical College of Wisconsin, 8701 Watertown Plank Rd, Milwaukee, WI 53226, United States of America

**Keywords:** 4D-MRI, Radiotherapy, In vivo, Visual biofeedback, MR-linac

## Abstract

**Background and Purpose:**

Managing respiratory motion is essential for effective radiotherapy in the abdominothoracic regions. Respiratory-correlated four-dimensional magnetic resonance imaging (4D-MRI) can provide accurate motion estimation to help define treatment volumes for adaptive radiotherapy. However, validating and comparing 4D-MRI sequences in vivo is challenging due to the presence of breathing variability. This study combines visual biofeedback (VBF) with 4D-MRI sequences to facilitate in vivo comparisons.

**Materials and Methods:**

Fourteen healthy volunteers and one patient were scanned on a 1.5 T Unity MR-linear accelerator (Elekta AB, Stockholm, Sweden) at two institutions. A radial stack-of-stars (SoS), a simultaneous multi-slice (SMS), and a Cartesian acquisition with spiral ordering (CASPR) 4D-MRI sequence were acquired. These acquisitions were performed without and with VBF based on an interleaved one-dimensional respiratory navigator (1D-RNAV) acquisition. Breathing variability across sequences was quantified using 1D-RNAV-derived breathing waveforms. Reconstructed 4D-MRI data were used to extract the motion amplitude, which was compared intra-volunteer across sequences and to the amplitudes of the breathing waveforms.

**Results:**

Breathing variability across sequences decreased by 37% (amplitude, *p*= 0.039) and 64% (period, *p*< 0.003), and the median intra-volunteer 4D-MRI-derived motion amplitude agreement improved from 3.5 mm to 1.8 mm (*p*= 0.064) across sequences due to VBF guidance. Four-dimensional MRI-derived amplitudes were smaller than breathing waveform amplitudes, with median differences of -31% (SoS), -17% (SMS), and -9% (CASPR). The average breathing waveform amplitude was 8% larger than instructed.

**Conclusions:**

This methodology enables in vivo comparisons of 4D-MRI sequences for adaptive radiotherapy, with guidance improving anatomical consistency and ensuring more reliable comparisons.

## Highlights

• Three different 4D-MRI sequences acquired in 14 healthy volunteers and 1 patient.
• Visual biofeedback improved breathing regularity across 4D-MRI sequences.
• Inter-sequence 4D-MRI-derived amplitude agreements improved from 3.5 mm to 1.8 mm.

## 1 Introduction

Respiratory motion management is crucial in abdominothoracic radiotherapy, as breathing is the main contributor to anatomical motion during treatment [1], [2]. Respiratory-correlated four-dimensional (4D) imaging sorts data retrospectively into three-dimensional (3D) volumes representing different respiratory phases. The current standard of care in radiotherapy is 4D computed tomography (4D-CT) for treatment planning. Additionally, 4D cone beam CT (4D-CBCT) may be used for position verification during treatment [3], [4].

The integration of magnetic resonance imaging (MRI) into radiotherapy has enhanced treatment precision, particularly in soft tissues. For respiratory motion management, a 4D-MRI can be acquired to serve two main purposes in radiotherapy: delineation and motion estimation. Important considerations include image contrast flexibility, geometric accuracy, spatial resolution, and acquisition and reconstruction times [5]. During MR-simulation, 4D-MRI is used to estimate breathing-induced tumor motion for treatment planning, and it is available as a commercial product [6], [7], [8]. Although vendor-provided 4D-MRI solutions on hybrid systems integrating an MRI scanner and a linear accelerator (MR-linac) are lacking [9], [10], in-house developed 4D-MRI has been shown to be effective for estimating motion on the day of treatment [11], [12].

Multiple 4D-MRI sequences have been developed, with most studies focusing on individual sequences and literature-based comparisons. Despite the availability of multiple sequences at some research centers, to our knowledge, a in vivo comparison has not been published. Such comparison is challenging due to the lack of a ground truth, as the anatomy in the thorax and abdomen moves due to respiration, which varies over time and may drift [13], [14]. When comparing 4D-MRI sequences, it is crucial to minimize breathing variability across scans, which can be achieved using visual biofeedback (VBF) [15], [16]. In MRI, a one-dimensional respiratory navigator (1D-RNAV) can be interleaved with image acquisition [17]. It is typically acquired at the liver-lung interface, providing a quantitative surrogate breathing waveform in the craniocaudal direction, which could be used as input for VBF [18].

In this study, we implement VBF with different 4D-MRI sequences used in radiotherapy. The primary goal is to enforce a similar breathing pattern across 4D-MRI acquisitions of the same subject, allowing for in vivo comparisons.

## 2 Materials and methods

### 2.1 Overview of 4D-MRI acquisition workflow

**Fig. 1:**
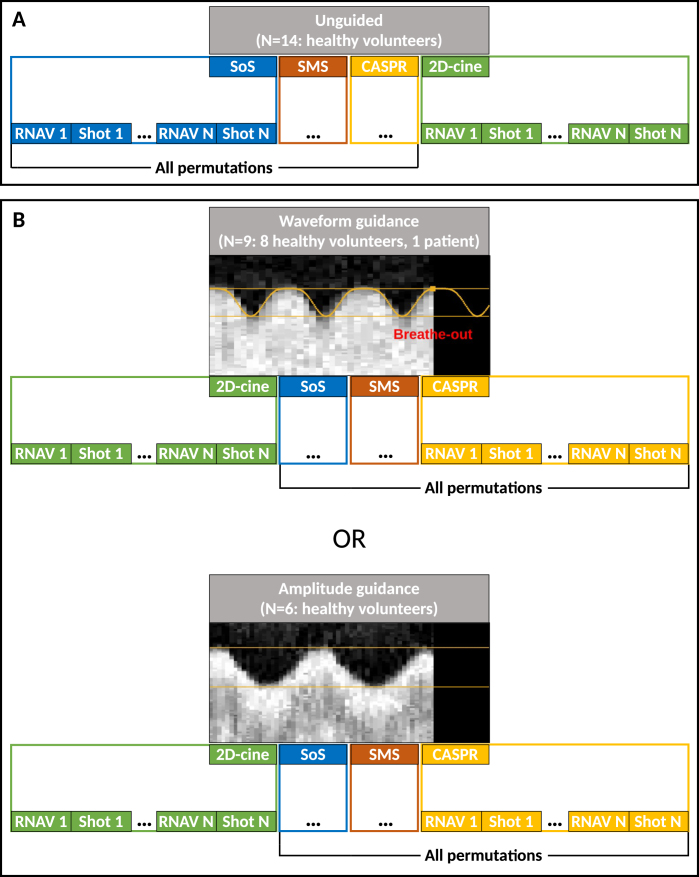
Schematic overview of the data acquisition. Data acquisition shots were interleaved with one-dimensional respiratory navigator (RNAV) shots. Stack-of-stars (SoS), simultaneous multi-slice (SMS), and Cartesian acquisition with spiral ordering (CASPR) 4D-MRI sequences were acquired without (A) and with (B) visual biofeedback guidance. Two-dimensional cine MRIs were acquired in between to estimate and validate subject-specific breathing parameters.

Fig. 1 shows the 4D-MRI acquisition methodology. To improve breathing regularity and harmonize breathing across sequences, 1D-RNAV data were acquired interleaved with 4D-MRI shots and displayed as VBF. The VBF hardware (i.e., mirror, in-room monitor, network connection) was similar to our previous study [18]. However, the VBF software was substantially modified to stream and display the acquired 1D-RNAV data (graphical user interface shown in Fig. 1B). Additionally, a lower latency was achieved by sending this data directly once acquired. First, 4D-MRI data were acquired in free-breathing without VBF as a reference. Second, an interleaved orthogonal sagittal/coronal 5.6 Hz two-dimensional (2D)-cine MRI sequence was acquired for 70 s to extract the subject-specific breathing parameters. The VBF software extracted a breathing waveform from the 1D-RNAV data, and peak detection defined individual breathing cycles used to derive the median amplitude and period. Two types of VBF were implemented: waveform guidance (VBF.w), which provided a Lujan motion waveform (cos${}^{4}$) with the subject-specific amplitude and period to guide both the amplitude and the period, and amplitude guidance (VBF.a), which used two horizontal lines to guide the amplitude. Third, a similar 2D-cine MRI acquisition was performed for 100 s with VBF, based on the automatically derived subject-specific period and/or amplitude, to familiarize the subjects with the VBF interface. Finally, 4D-MRI scans with VBF were acquired using the automatically derived or adjusted (based on the subjects’ feedback) subject-specific breathing parameters.

### 2.2 Data acquisition

Data were acquired in twelve healthy volunteers (age 24–36 years, weight 56–85 kg, male:female 9:3) at University Medical Center Utrecht (UMCU), and in two healthy males (age 44–54 years, weight 84–86 kg) and one male patient (age 83 years, weight 82 kg, oligometastatic pancreatic cancer to the liver) at Froedtert/Medical College of Wisconsin (Froedtert/MCW). Acquisitions were performed in arms up position on a 1.5 T Unity MR-linac (Elekta AB, Stockholm, Sweden) and all subjects provided written informed consent before participation.

The 1D-RNAV acquired 72 mm of data at 1 mm resolution along the craniocaudal direction at the liver-lung interface, and was interleaved with three different 4D-MRI sequences: a fat-suppressed T2/T1-weighted radial stack-of-stars (SoS) acquisition [11], a T2-weighted simultaneous multi-slice (SMS) acquisition [19], and a T1-weighted Cartesian acquisition with spiral profile ordering (CASPR) modified from Prieto et al. [20], [21]. Among the different contrast-weighting options, these are the most commonly used in clinical practice at Froedtert/MCW (SoS), in research at UMCU (SMS), and in research at The Institute of Cancer Research/The Royal Marsden Hospital (CASPR). Scan parameters are summarized in Supplementary Table S1. The field-of-view (FOV) and voxel sizes were matched as best as possible and the scans were tailored to a five-minute acquisition time. The three 4D-MRI scans were acquired in six unique permutations, each performed with either VBF.w or VBF.a, resulting in twelve distinct MRI sessions in Utrecht. At Froedtert/MCW, the three subjects were guided with VBF.w. Patient data were acquired during a pre-treatment imaging study with the unguided 4D-MRI acquisitions removed from the protocol due to time restrictions.

### 2.3 Offline 4D-MRI reconstruction and patient data evaluation

The 4D-MRI data were retrospectively sorted into ten respiratory bins using amplitude binning that accounted for hysteresis [19], [22] and corrected for gradient non-linearities [23]. For the SMS sequence, data were sorted in image domain, while for the SoS and CASPR sequences, data were sorted in k-space domain. All processing was performed on a local reconstruction workstation (36 cores, 256 GB RAM), further reconstruction details are available in Supplementary material A.

For the patient data, the gross tumor volume (GTV) was delineated by an experienced senior physician (WH) on derived mid-position (time-weighted average) images [24]. In addition, the physician scored tumor border visibility based on effort required for delineation, using a four-point scale: “very poor”, “poor”, “good”, and “excellent”. Contouring and analyses were performed in MIM version 7.2.9 (MIM Software Inc., Cleveland, OH). Analyses involved calculating the Dice similarity coefficient (DSC) and the mean surface distance (MSD) of pairwise comparisons between delineations.

### 2.4 Inter-sequence breathing variability

Breathing variability was analyzed using the coefficient of variation (CV) in the breathing amplitude and period. Individual breathing cycles were extracted from 1D-RNAV-derived breathing waveforms per acquisition using peak detection. The standard deviation and mean for the amplitude and period were then calculated, followed by the calculation of the CV (Eq. (1)):

(1) $${\text{CV}}_{\text{a,b}}=\frac{\sigma_{\text{a,b}}}{\mu_{\text{a,b}}}$$

where a∈{Unguided,Guided}, b∈{Amplitude,Period}.

A comparison across sequences (inter-sequence) was performed by calculating the absolute difference of CV values (|ΔCV|) for unguided and guided acquisitions separately. A |ΔCV| close to zero indicates similar motion variability across different sequences.

### 2.5 1D-RNAV latency and sensitivity

The latency between acquiring and displaying the 1D-RNAV data was quantified using a motion phantom (Quasar MRI4D, Modus Medical Devices Inc., London ON), by calculating the phase difference between phantom-reported positions and positions derived from displayed 1D-RNAV data [25].

Furthermore, the phantom was used for a sensitivity analysis of the 1D-RNAV-derived breathing waveforms. The 4D-MRI sequences were acquired with and without the 1D-RNAV FOV and imaging FOV overlapping while the phantom was moving with 25 mm peak-to-peak. Phantom-reported positions were interpolated to the 1D-RNAV acquisition time points. Amplitudes were then derived from both these phantom positions and 1D-RNAV data.

### 2.6 Comparison of motion amplitude

The 4D-MRI motion amplitude was derived using template matching (normalized cross-correlation) on the sorted 4D-MRIs at the 1D-RNAV acquisition location. The motion amplitudes of the 4D-MRIs were compared to the motion amplitudes of the breathing waveforms and the amplitudes used for guidance.

### 2.7 Self-sorting signal vs. 1D-RNAV-derived breathing waveform

For the SoS and CASPR sequences, data were sorted based on self-navigation surrogate signals further described in Supplementary material A. These self-sorting signals were compared to the 1D-RNAV-derived breathing waveforms by calculating the Pearson correlation and the root mean square deviation (RMSD) between the two types of surrogate signals. The 1D-RNAV-derived breathing waveforms were normalized between 0 and 1 using 98% of the data minimizing the peak-to-peak amplitude. In addition, the phase shift between the 1D-RNAV-derived breathing waveforms and self-sorting signals was determined and accounted for.

### 2.8 Statistical tests

The differences in breathing variability and motion amplitudes were quantified with a two-sided Wilcoxon signed rank test with a significance level of 5%.

## 3 Results

Data acquisition was 99% successful, with adequate quality 1D-RNAV data in 86 out of 87 4D-MRI acquisitions. For the patient, 1D-RNAV data acquired during the SoS sequence lacked the quality necessary to guide the breathing. The latency between acquiring and displaying the VBF was 50 ± 18 ms. The 2D-cine MRI acquisition was sufficient to familiarize subjects to the VBF and to verify if the amplitude (VBF.w/VBF.a) and period (VBF.w) were accurately set.

The 4D-MRI acquisition times were set to five minutes, resulting in 897, 810, and 1472 acquisition shots for the SoS, SMS, and CASPR acquisitions, respectively. Multiplying these shots by their corresponding turbo factors (Supplementary Table S1), a total of 49,335, 47,790, and 47,104 k-space readouts were performed, agreeing within 5%. Reconstructing 4D-MRIs from the SMS data took in the order of seconds as images were reconstructed by the host reconstructor. For the 3D based methods, reconstruction times were around 500 s for SoS and 200 s for CASPR. Performing gradient non-linearity correction took 3 s.

**Fig. 2:**
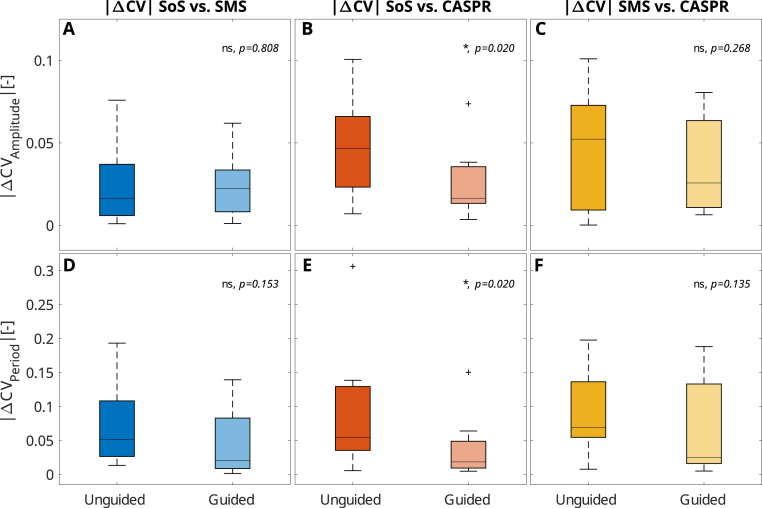
The absolute difference in the coefficient of variation (|ΔCV|) of breathing amplitude (A–C) and period (D–F), compared between the stack-of-stars (SoS), simultaneous multi-slice (SMS), and Cartesian acquisition with spiral ordering (CASPR) sequences for unguided and guided acquisitions. Values closer to zero indicate higher breathing similarity across sequences.

### 3.1 Breathing variability across 4D-MRIs

Breathing was statistically significantly harmonized across sequences, as the median $\left|\Delta {\text{CV}}_{\text{Amplitude}}\right|$ and $\left|\Delta {\text{CV}}_{\text{Period}}\right|$ decreased by 37% (*p* = 0.039) and 64% (*p* < 0.003), respectively. Fig. 2 shows the |ΔCV| values of inter-sequence CV comparisons for the healthy volunteers. Pair-wise comparisons revealed statistically significant (*p* = 0.020) reductions in $\left|\Delta {\text{CV}}_{\text{Amplitude}}\right|$ and $\left|\Delta {\text{CV}}_{\text{Period}}\right|$ between the SoS and CASPR acquisitions, but not for the other sequence pairings.

Based on amplitudes derived from breathing waveforms, the median (min:max) amplitude differences within each volunteer across different 4D-MRI scans decreased from 2.4 (0.0:11.8) mm to 2.1 (0.1:13.3) mm (*p* = 0.365) when data were acquired with VBF. Similarly, the intra-volunteer 4D-MRI-derived amplitude differences decreased from 3.5 (0.0:15.8) mm to 1.8 (0.0:14) mm (*p* = 0.064) when comparing data acquired without and with VBF, respectively.

### 3.2 1D-RNAV sensitivity

Supplementary Table S2 summarizes the amplitudes derived from phantom data. Absolute differences within 0.6 mm (SoS), 0.8 mm (SMS), and 1.0 mm (CASPR) were found between the mean amplitudes of the phantom waveforms and 1D-RNAV-derived breathing waveforms. Comparing acquisitions without and with overlapping FOVs revealed differences of 0.6 mm (SoS), 0.1 mm (SMS), and 0.0 mm (CASPR). The largest inter-sequence difference was 1.0 mm (SoS with overlap vs. SMS with overlap).

### 3.3 Guidance signal quality

**Fig. 3:**
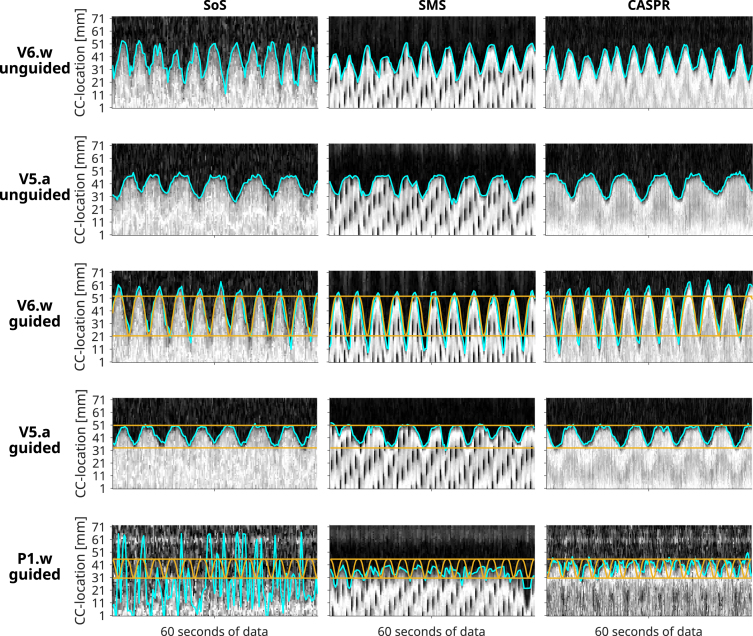
Examples of three subjects of the one-dimensional respiratory navigator data at the liver-lung interface (black is lung, white is liver) with the guidance in amber superimposed for the stack-of-stars (SoS), simultaneous multi-slice (SMS), and Cartesian acquisition with spiral ordering (CASPR) sequences. Extracted breathing waveforms are indicated in cyan. Guidance improved the breathing regularity. Note the signal saturation effects in the navigator data for the SMS sequence as a result of the axial image acquisition. For the patient (P1.w), no unguided 4D-MRI acquisitions were performed and the navigator during the SoS acquisition lacked quality required for guidance.

Fig. 3 shows examples of 1D-RNAV data acquired in vivo during unguided and guided acquisitions, with an example using waveform guidance (V6.w) where the amplitude was higher than instructed, and an example of amplitude guidance (V5.a) demonstrating high consistency. In general, the 1D-RNAV data acquired during the CASPR sequence had the highest quality in terms of contrast between signals acquired in the lung and the liver. The CASPR sequence had the shortest shot duration and therewith shorter repetition times for the VBF compared to the other sequences. This was also noted by most volunteers, who preferred the guidance during the CASPR acquisition. The axial SMS acquisition interleaved with the 1D-RNAV resulted in RNAV data with saturation effects (i.e., black saturation stripes in the liver).

The 1D-RNAV data acquired during the SoS sequence in patient P1.w had no contrast between the lung and the liver. As a result, the patient was unable to follow these guidance instructions and analysis of the 1D-RNAV data was not possible.

**Fig. 4:**
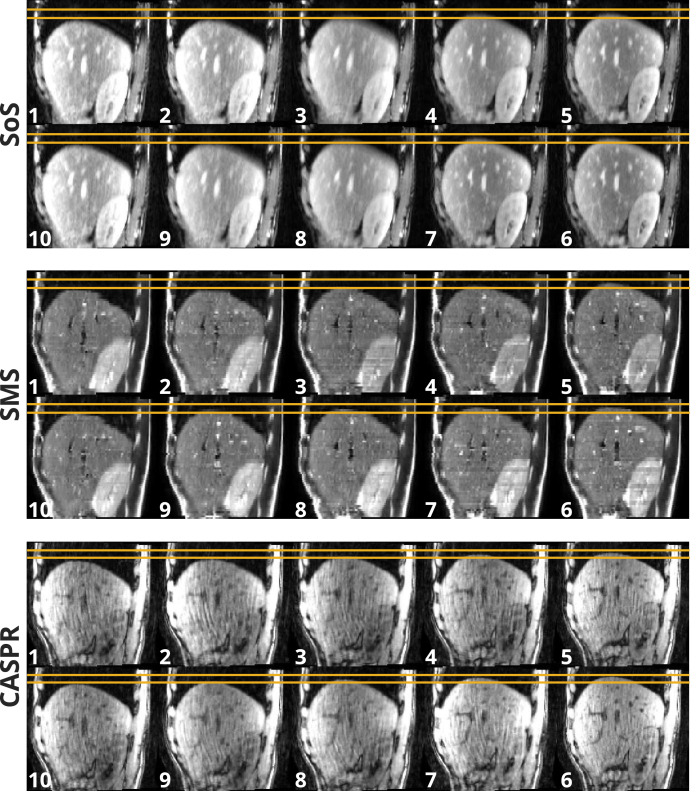
Examples of respiratory correlated 4D-MRIs of volunteer V3.w. Data were sorted into ten respiratory phases that accounted for hysteresis, where end-inhale is represented by phase 1 and end-exhale by phase 6. The amber lines indicate the amplitude window that was used as visual biofeedback during the data acquisition.

**Fig. 5:**
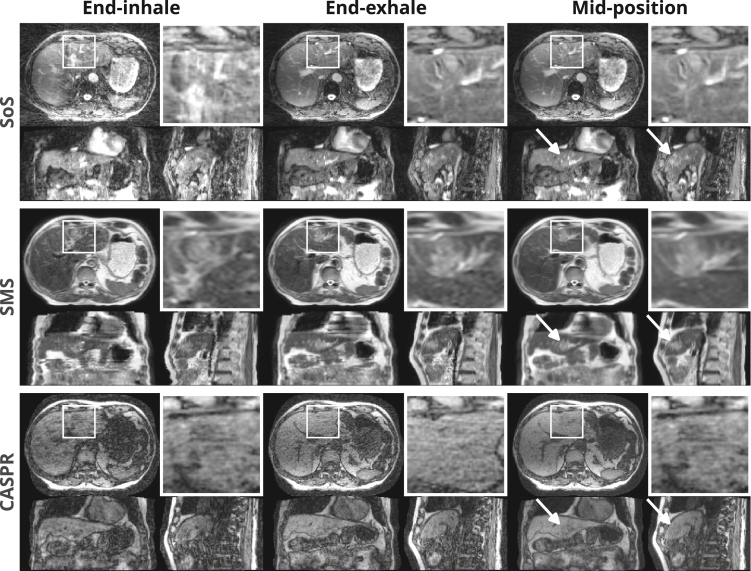
End-inhale, end-exhale, and mid-position images of patient P1.w shown in three orthogonal views through the tumor location. A zoomed-in box is provided in the axial orientation, and arrows indicate the tumor in the other orientations.

### 3.4 Image quality

Fig. 4 shows a 4D-MRI example of volunteer V3.w with the superimposed guidance amplitudes as reference. Adding 1D-RNAV acquisitions did not have a visible impact on the image quality. Fig. 5 shows the end-inhale, end-exhale, and mid-position images of patient P1.w at the tumor location for the three 4D-MRI sequences. According to the physician, the tumor was visible in the acquired data for all sequences, with the SoS and SMS mid-position images showing “good” tumor border visibility, whereas the CASPR mid-position image had a “poor” tumor border visibility. Pairwise comparisons of GTV delineations had DSC values of 0.50–0.75 and MSD values of 1.4–3.3 mm. Supplementary Video S1 and Video S2 demonstrate the respiratory phases of V3.w and P1.w for the three sequences in a continuous video.

### 3.5 Motion amplitude consistency

**Fig. 6:**
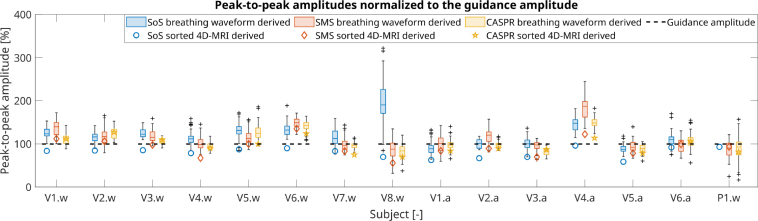
Normalized peak-to-peak amplitudes extracted from one-dimensional respiratory navigator-derived breathing waveforms and sorted 4D-MRIs acquired with radial stack-of-stars (SoS), simultaneous multi-slice (SMS) and Cartesian acquisition with spiral ordering (CASPR) sequences with guidance. The box plots show the distributions of amplitudes derived from individual breathing cycles. Note that the 1D-RNAV data acquired during the SoS scan of patient P1.w was of insufficient quality for analysis (i.e., no box plot).

Fig. 6 shows the relative peak-to-peak amplitudes extracted from the 1D-RNAV-derived breathing waveforms and 4D-MRI data for the guided experiments normalized to the guidance amplitude. On average, smaller amplitudes were derived from the 4D-MRI data compared to the breathing waveforms. Median (2.5th:97.5th percentile) differences of -31 (-68:-5)%, -17 (-45:28)%, and -9 (-35:26)% were observed for the guided SoS (*p* < 0.001), SMS (*p* < 0.001), and CASPR (*p* = 0.013) acquisitions, respectively. The SMS and CASPR values include the patient data as well. The subjects breathed on average with a larger amplitude than requested, with median (2.5th:97.5th percentile) differences of 8 (-32:86)% (*p* < 0.003) between guidance and 1D-RNAV-derived amplitudes. V6.w and V4.a had a notable larger motion amplitude than the provided VBF, which is also visible in Fig. 3 for V6.w. Breathing amplitudes are summarized in Supplementary Table S3.

### 3.6 Self-sorting signal vs. 1D-RNAV-derived breathing waveform

For the SoS and CASPR data acquired at UMCU, high Pearson correlations were observed between the self-sorting signals and 1D-RNAV-derived breathing waveform, with median values ranging from 0.95 to 0.98 for both unguided and guided experiments. Similarly, comparable RMSD values were found across both SoS and CASPR signals, with median values ranging from 0.08 to 0.11, reflecting both unguided and guided conditions.

For the data acquired at Froedtert/MCW, the 1D-RNAV data were noisier, especially for the SoS data acquisition. The correlations between the self-sorting signals and breathing waveforms were between 0.43–0.89 for the SoS sequence and between 0.75–0.97 for the CASPR sequence. RMSD values were higher, ranging from 0.10–0.43 for SoS and 0.08–0.22 for CASPR.

## 4 Discussion

The developed methodology combined 4D-MRI acquisitions with a 1D-RNAV acquisition for VBF. The VBF was displayed with a latency of 50 ms and 100 s was sufficient to train the subjects. The results demonstrated improved inter-scan breathing regularities and agreements in 4D-MRI amplitudes (from 3.5 mm to 1.8 mm). This methodology improves the consistency and reliability of 4D-MRI sequences, enabling more accurate in vivo comparisons of 4D-MRI sequences suitable for abdominothoracic radiotherapy on the MR-linac.

Acquisition and reconstruction times are critical factors in evaluating the suitability of 4D-MRI sequences for radiotherapy. Although k-space readouts differed by only 5% for the five-minute acquisitions, reconstruction times varied substantially. The SMS images, reconstructed by the MR host, were sorted within seconds. In contrast, CASPR and SoS reconstructions took over 3 and 8 min, respectively, using our reconstruction workstation. Additional processing times will be involved during treatment to derive 3D mid-position images compatible with the treatment planning system [11], [12]. While manageable for MR-simulation, these times emphasize the need for high-performance workstations for integrating these sequences into online radiotherapy workflows. For the SoS and CASPR sequences, shortening acquisition times can lead to faster reconstruction, but it requires advanced reconstruction methods to mitigate more severe undersampling artifacts. Alternatively, deep learning approaches could reconstruct 4D-MRIs in under 30 s [26], [27] or even subsecond [28].

VBF based on a 1D-RNAV acquisition reduced intra- and inter-scan breathing variability, enabling effective in vivo 4D-MRI sequence comparisons. The 1D-RNAV data of the SMS sequence showed saturation effects, which impacted VBF performance when it occurred at the liver-lung interface. Despite this, volunteers were able to ignore this temporary misinformation. Displaying the acquired 1D-RNAV data provided the subjects with contextual information about the motion, avoiding misleading derived positions affected by these saturation effects [18]. The saturation also affected the breathing waveform extraction, but no systematic effects were observed after removing outliers. The CASPR acquisition provided good lung-liver contrast and a higher update frequency, making it easier for volunteers to adjust their breathing.

Choosing the 1D-RNAV for VBF made the quality and temporal resolution sequence-dependent. Ideally, internal breathing motion could be estimated independently of the MRI sequence using methods like noise variance, respiratory bellows, or an additional independent radiofrequency signal [29], [30], [31]. However, these surrogates typically provide breathing phase rather than reliable breathing amplitude information, which is crucial for accurate tumor motion estimation in radiotherapy. The high correlations observed between the 1D-RNAV-derived breathing waveforms and 4D-MRI self-sorting signals at UMCU suggest that these signals are reliable for sorting and potentially for VBF (assuming real-time processing is feasible), though using them for VBF introduces sequence-dependent latency. While no difference was observed between data acquired at two different systems at UMCU, the noisier 1D-RNAV data acquired at Froedtert/MCW, potentially attributable to radiofrequency interference among other factors, underscores the need for robust methods.

Data were acquired in one patient as proof-of-principle to investigate tumor visibility across different contrasts of the 4D-MRI sequences. Compared to healthy volunteers, the patient was positioned further cranially inside the MR-linac bore for the regular imaging study, moving the imaging and RNAV FOVs further away from isocenter, which might have degraded the SoS 1D-RNAV data quality. Regarding tumor visibility, the physician observed that the tumor was less visible in the T1-weighted CASPR data. Acquiring different images contrasts during MR-simulation can help identify the optimal contrast for tumor visibility [11].

Another key aspect of a suitable 4D-MRI is accurate motion estimation. VBF nearly halved the inter-sequence 4D-MRI-derived motion amplitude differences from 3.5 mm to 1.8 mm. The CASPR data showed best agreements between 4D-MRI-derived and RNAV-derived amplitudes. However, these 4D-MRI-derived amplitudes were still smaller on average (Fig. 6). This can partly be explained by the 4D-MRIs representing an average over five minutes. Additionally, data binning reduces the amplitude, as the end-inhale and end-exhale phases represent average positions rather than the motion extremes. Consequently, treatment plans may require an extra margin to account for the actual anatomical motion. This effect depends on the binning implementation and should therefore be determined per protocol. The interleaved 1D-RNAV acquisition provides in vivo motion information, which is valuable for optimizing the regularization parameters of 4D-MRI sequences. While our approach assumes compatibility between the 1D-RNAV and the 4D-MRI sequence, alternative methods providing in vivo motion could be considered.

The results of this comparison methodology are promising, demonstrating improved breathing regularity across 4D-MRI sequences. This method provides an invaluable tool for sequence development, acceptance testing, and potentially for quality assurance in radiotherapy. Although the study showed positive results in healthy volunteers and one patient, future research should explore sequence-independent VBF with sufficient temporal resolution to further enhance in vivo breathing regularity for comparisons.

## CRediT authorship contribution statement

**Katrinus Keijnemans:** Conceptualization, Data curation, Formal analysis, Investigation, Methodology, Software, Validation, Visualization, Writing – original draft. **Tim Schakel:** Conceptualization, Methodology, Software, Resources, Writing – review & editing. **Bastien Lecoeur:** Conceptualization, Resources. **Pim T.S. Borman:** Conceptualization, Resources, Visualization, Writing – review & editing. **William A. Hall:** Conceptualization, Formal analysis, Resources. **Bas W. Raaymakers:** Conceptualization, Supervision, Writing – review & editing. **Andreas Wetscherek:** Conceptualization, Resources, Writing – review & editing. **Eric S. Paulson:** Conceptualization, Resources, Writing – review & editing. **Martin F. Fast:** Conceptualization, Funding acquisition, Methodology, Project administration, Supervision, Validation, Visualization, Writing – review & editing.

## Declaration of competing interest

The authors declare the following financial interests/personal relationships which may be considered as potential competing interests: University Medical Center Utrecht, The Institute of Cancer Research (ICR), The Royal Marsden Hospital (RMH), and Medical College of Wisconsin (MCW) are members of the MR-Linac Consortium with industrial partners Elekta and Philips. BL and AW: ICR and RMH receive research support from Elekta AB and Philips Healthcare. WH and EP: MCW receives research support from Elekta Instruments AB, Siemens Healthineers, MIM Software, RaySearch Laboratories, and Philips Healthcare. WH: Consultant Aktis Oncology, Co-founder/Shareholder/Consultant for Sonoptima Inc

## Appendix A Supplementary data

MMC S1 Acquisition and reconstruction methods with a summary of derived breathing waveform amplitudes.
