# Supplementary material for: Enabling in vivo comparisons of different four-dimensional magnetic resonance imaging sequences for radiotherapy guidance using visual biofeedback
Source: Phys Imaging Radiat Oncol. 2025 Aug 5;35:100815. doi: 10.1016/j.phro.2025.100815 (PMC12347986; doi:10.1016/j.phro.2025.100815)
Supplement: MMC S1 — Acquisition and reconstruction methods with a summary of derived breathing waveform amplitudes. [file mmc1.pdf]

4D-MRI scan parameters

Table S1: Summary of the scan parameters for the stack-of-stars (SoS), simultaneous multi-slice (SMS), and Cartesian acquisition with spiral ordering (CASPR) 4D-MRI sequences.

	SoS	SMS	CASPR
Sequence type [-]	Gradient echo	Spin echo	Gradient echo
k-space trajectory [-]	Radial	Cartesian	Cartesian spiral
Image contrast [-]	T2/T1-weighted	T2-weighted	T1-weighted
Fat suppression [-]	SPAIR	No	No
Orientation [-]	Axial	Axial	Coronal
Dimensions (LR×AP×CC) [mm^3]			
Field-of-view	420×420×210	420×420×210	420×300×210
Acquired voxel size	1.64×1.64×3.50	2.00×3.00×3.50	1.64×1.64×1.75
Reconstructed voxel size	1.64×1.64×1.75	1.64×1.64×3.50	1.64×1.64×1.75
Number of slices [-]	120	60	120
In-plane parallel imaging factor [-]	1	1.5	1
Partial Fourier factor [-]	0.7	0.625	1
Number of shots [-]	897	810	1472
Flip angle [°]	50	90/110	15
Echo time / Repetition time [ms]	4.2/2.1	77/11026	5.6/2.5
Turbo factor [-]	55	59	32
Shot duration [ms]	336	353	206
Scan time [s]	303	298	306

A. 4D-MRI reconstruction details

A.1. Respiratory sorting

For the data binning, the end-exhale and end-inhale bin sizes were half the size of the eight bins describing inhalation and exhalation, to minimize imbalances in the data distribution over the different phases.

A.2. Surrogate signal and image reconstruction

For the SMS data, the default MRI host reconstructor was used to reconstruct raw k-space data into images that were written into DICOM format. These DICOM images were sorted based on breathing waveforms that were derived from the 1D-RNAV data using edge-detection. An outlier detection filter was used to remove incorrect edge-detections.

For the SoS and CASPR data, the raw k-space data were transferred from the host scanner to a local reconstruction workstation (36 cores Intel Xeon Gold 2.60GHz, 256GB RAM). For the SoS data, the acquired k-space data with partial Fourier were zero-filled. The k-space data were sorted based on self-navigation by extracting a motion surrogate signal in the cranial-caudal direction, as this is the main direction of breathing-induced motion. For the SoS data, this involved taking projections along the cranial-caudal direction through the center of k-space for each acquisition shot. For the CASPR data, the readout direction was in the cranial-caudal direction. Because of the spiral-out phase encodes, the first readout of every shot acquired the center of k-space and was therefore used as motion surrogate signal for self-sorting. Self-sorting signals were derived using a principal component analysis and then scaled between 0 and 1, with 98% of the data used to minimize outliers in the self-sorting signal. The 2% of data outside the range were set to their respective minimum or maximum. Based on the sorted data into respiratory bins, SoS data were reconstructed using parallel imaging with wavelet (spatial) and low-dimensional subspace (temporal) constraints using a linear conjugate gradient solver, whereas the CASPR data were reconstructed using parallel imaging and compressed sensing with wavelet (spatial) and total variation (temporal) constraints using the BART toolbox. For the SoS reconstructions,

the k-space data per phase were scaled based on the sum of squares k-space magnitude of the end-exhale phase to prevent signal intensity fluctuation over different phases resulting from the amplitude binning. For the CASPR reconstructions, the spatial regularization parameter was scaled based on the sum of squares k-space magnitude to obtain consistent reconstructions over the different data sets.

Sensitivity analysis

Table S2: Amplitudes (mean ± standard deviation) derived from the interpolated phantom waveform and the acquired one-dimensional respiratory navigator (1D-RNAV) data for the stack-of-stars (SoS), simultaneous multi-slice (SMS), and Cartesian acquisition with spiral ordering (CASPR) sequences without and with overlapping field-of-view (FOV).

**Imaging sequence and 1D-RNAV FOV**	**Interpolated phantom waveform amplitude [mm]**	**1D-RNAV-derived breathing waveform amplitude [mm]**
SoS without overlap	24.9 ± 0.1	24.3 ± 0.5
SoS with overlap	24.9 ± 0.1	24.9 ± 0.6
SMS without overlap	24.7 ± 0.3	24.0 ± 0.2
SMS with overlap	24.7 ± 0.3	23.9 ± 0.8
CASPR without overlap	25.0 ± 0.0	24.0 ± 0.2
CASPR with overlap	25.0 ± 0.0	24.0 ± 0.3

1D-RNAV-derived breathing waveform amplitudes

Table S3: One-dimensional respiratory navigator (1D-RNAV)-derived amplitude (mean ± standard deviation) for the stack-of-stars (SoS), simultaneous multi-slice (SMS), and Cartesian acquisition with spiral ordering (CASPR) sequences. Also the visual biofeedback (VBF) guidance amplitude is provided.

Subject	Amplitude [mm]						
	Unguided				Guided		
	SoS	SMS	CASPR	VBF guidance	SoS	SMS	CASPR
V1.w	23.6 ± 4.5	21.7 ± 3.3	21.1 ± 4.2	25.0	31.3 ± 2.9	33.9 ± 4.3	27.7 ± 2.9
V2.w	16.1 ± 2.8	17.6 ± 3.4	17.8 ± 2.1	16.5	19.0 ± 1.8	19.4 ± 3.1	20.5 ± 2.0
V3.w	10.7 ± 1.4	12.6 ± 1.6	13.2 ± 0.8	14.3	17.9 ± 1.5	17.0 ± 2.1	15.1 ± 0.9
V4.w	17.7 ± 2.2	17.9 ± 2.9	18.4 ± 1.8	15.5	17.5 ± 2.1	15.6 ± 2.1	14.7 ± 1.4
V5.w	10.3 ± 1.8	11.1 ± 1.5	13.0 ± 1.9	14.0	18.3 ± 2.4	16.0 ± 2.2	17.8 ± 2.6
V6.w	30.9 ± 5.9	25.3 ± 4.9	25.6 ± 3.8	31.0	41.3 ± 4.6	46.1 ± 3.5	43.9 ± 3.8
V7.w	26.0 ± 5.7	19.2 ± 3.3	24.8 ± 4.3	21.0	24.1 ± 3.8	20.8 ± 3.3	20.2 ± 1.5
V8.w	18.2 ± 3.5	14.9 ± 3.0	13.7 ± 2.8	25.0	24.7 ± 5.8	11.0 ± 2.5	10.5 ± 2.2
V1.a	13.5 ± 2.5	14.8 ± 2.6	17.4 ± 5.0	25.0	22.6 ± 3.5	25.0 ± 4.6	24.4 ± 2.9
V2.a	16.5 ± 3.6	26.1 ± 5.0	21.9 ± 3.6	23.5	23.6 ± 2.3	27.5 ± 3.9	23.1 ± 1.6
V3.a	24.0 ± 3.7	17.5 ± 2.9	16.8 ± 1.4	20.0	20.0 ± 2.7	19.2 ± 2.1	16.3 ± 1.6
V4.a	13.6 ± 3.1	20.7 ± 4.4	23.8 ± 7.1	20.0	29.3 ± 3.1	36.9 ± 5.1	29.9 ± 2.2
V5.a	18.9 ± 2.3	20.2 ± 3.3	17.4 ± 3.0	17.8	15.8 ± 1.7	16.9 ± 2.4	15.9 ± 1.7
V6.a	30.5 ± 3.9	27.4 ± 5.2	26.2 ± 6.6	24.7	27.1 ± 4.2	24.0 ± 3.9	26.3 ± 4.4
P1.w	NA	NA	NA	15.0	NA	13.1 ± 2.9	13.6 ± 4.2
