# Supplementary figures and images for: Enabling in vivo comparisons of different four-dimensional magnetic resonance imaging sequences for radiotherapy guidance using visual biofeedback
Source: Phys Imaging Radiat Oncol. 2025 Aug 5;35:100815. doi: 10.1016/j.phro.2025.100815 (PMC12347986; doi:10.1016/j.phro.2025.100815)

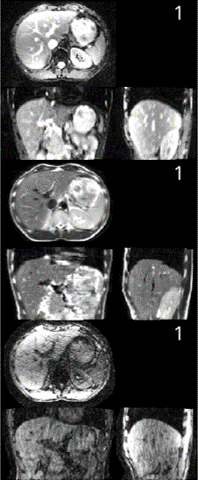

Supplement: Video S1 — . [file mmc2.gif]

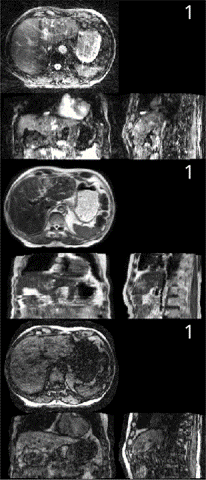

Supplement: Video S2 — . [file mmc3.gif]
